# A Rare Case of Emphysematous Osteomyelitis of Femur in an Intravenous Drug User

**DOI:** 10.7759/cureus.16782

**Published:** 2021-07-31

**Authors:** Imroz Singh Sachdev, Neeru Tomer, Sarath Bethapudi, Sarv Priya, Swapndeep Atwal

**Affiliations:** 1 Radiology, County Durham and Darlington NHS Foundation Trust, Durham, GBR; 2 Radiology, County Durham and Darlington NHS Foundation Trust, Darlington, GBR; 3 Radiology, University of Iowa Hospitals and Clinics, Iowa City, USA

**Keywords:** emphysematous osteomyelitis, osteomyelitis diagnosis, ct (computed tomography) imaging, msk radiology, intravenous drug user, anaerobic osteomyelitis

## Abstract

Emphysematous osteomyelitis (EO) is a rare condition characterized by the appearance of gas locules within the bone on imaging, usually as a result of anaerobic bacterial infection. We present the case of a 46-year-old known intravenous (IV) drug user who was admitted to the emergency department with intractable pain in the right groin. He was febrile with elevated white cell count and C-reactive protein. He underwent an X-ray and CT of the pelvis which demonstrated intraosseous gas in the proximal right femur. A diagnosis of EO was made radiologically, allowing for prompt antibiotic treatment and a plan for surgical debridement. There are only a handful of published cases of EO in the literature, only one of which has described IV drug use as the underlying factor.

## Introduction

Emphysematous osteomyelitis (EO) is a rare form of infection characterized by gas locules within the bone. It is potentially life-threatening if not diagnosed and treated in time. Imaging plays a vital role in establishing the diagnosis and a CT scan is often required to guide timely management.

## Case presentation

A 46-year-old male presented to the Emergency Department with a history of fever and intractable pain in the right groin. The patient was a known IV drug user without any other underlying medical condition. He had multiple past hospital admissions for various reasons. The inflammatory markers including C-reactive protein and white cell count were elevated.

**Figure 1:**
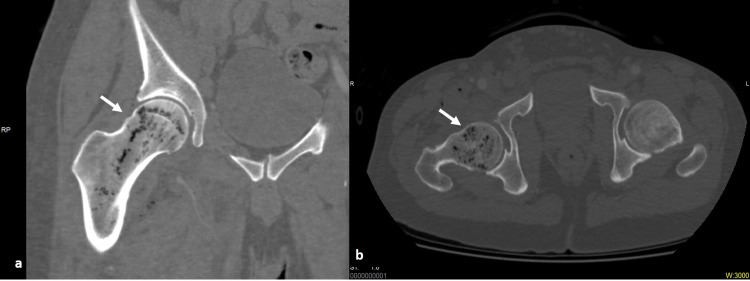
Intraosseous gas locules within the proximal right femur on CT bone window (arrows), coronal (a) and axial (b).

**Figure 2:**
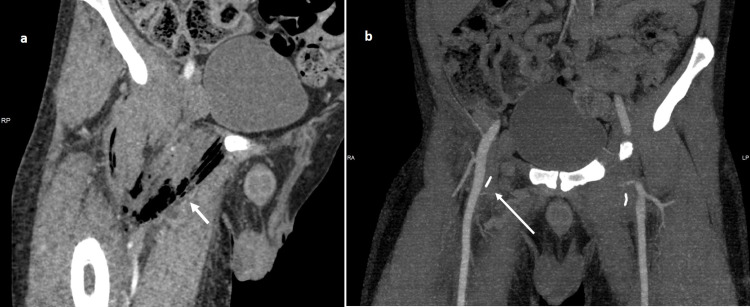
(a) Intramuscular gas in the right thigh (short arrow), (b) Intact femoral arteries. Linear metal objects (broken needles) embedded in the soft tissue of the groin on both sides (long arrow).

He was referred to the Radiology Department for CT angiography of the lower limb arteries to exclude pseudoaneurysm because of frequent needle usage for drug administration. On CT, multiple air locules were noted within the proximal femur (Figures 1a, b), the surrounding joint space and soft tissue including the muscles and the intermuscular planes (Figure 2a).

The femoral vessels were intact. Linear metallic foreign objects were noted in the soft tissue of the groin on both sides, consistent with embedded needles (Figure 2b).

**Figure 3:**
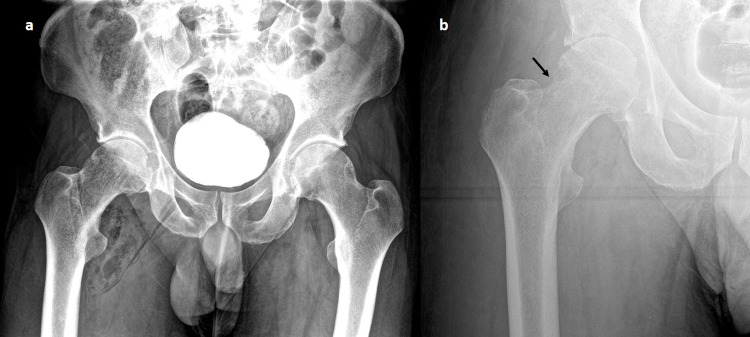
(a) Initial plain film showing gas within the femoral head and the soft tissue. X-ray was acquired shortly after the CT, thus contrast within the urinary bladder. The needles in the groin are very subtle. (b) Plain film eight weeks later shows the resolution of the gas. A subtle cortical defect is seen in the lateral aspect of the femoral neck secondary to the infection (arrow).

An X-ray pelvis was performed on the same day as a baseline for future follow-ups, which showed multiple radiolucent areas in the soft tissue around the right hip (Figure 3a). A preliminary diagnosis of emphysematous osteomyelitis was made based on CT findings. MRI was deemed unsuitable for this patient due to the risk of injury from embedded needles close to the femoral vessels.

The patient was subsequently transferred to a tertiary care hospital for surgical debridement. The culture of the debrided tissue grew anaerobic organisms, namely Finegoldia magna, a Gram-positive coccus and Actinomyces europaeus, a Gram-positive bacillus on enriched medium. He was treated with a long course of Clindamycin.

On follow-up after eight weeks, the patient was afebrile and the inflammatory markers were within normal limits. On X-ray, the intraosseous gas had resolved, though subtle cortical changes from osteomyelitis were evident (Figure 3b).

## Discussion

In 1981, intraosseous gas was first described as a sign of osteomyelitis by Ram et al. [1]. The presence of intraosseous gas strongly indicates osteomyelitis in an appropriate clinical setting where no other feasible explanation for the gas is present, such as trauma, penetrating wound or recent surgery.

Sulyma et al. [2] reviewed 49 cases of EO until 2020. About 85% of cases were associated with an underlying condition responsible for reduced immunity, the commonest being diabetes and malignancy. Other causes include alcohol abuse [3], sickle cell anemia [4] and steroid treatment. One case report described IV drug use in a patient with other comorbidities of hepatitis C and relapsed acute myeloid leukemia (AML) [5]. Luey et al. [6] reviewed 25 reported cases of EO until 2011 observed underlying comorbidities in about 75% of the cases.

Involvement of both axial and appendicular skeleton by EO have been reported, including lumbar vertebrae, femur [7], pelvic bones, sacrum, tibia, fibula and the foot bones [8]. Even though the gas in the vertebral bodies is usually non-infectious in etiology, likely secondary to degenerative changes [6], the gas in the appendicular skeleton is considered pathognomic for infection.

Imaging plays a crucial role in diagnosis, notably as the clinical differential for groin pain are wide. Plain radiographs are the mainstay of imaging in acute setting; however, diagnosis of intraosseous gas is challenging in them. CT, on the other hand, is highly sensitive in demonstration of the gas locules and is supported by the review of published case reports [2]. MRI is generally considered as the appropriate second-line imaging modality after plain radiographs in suspected cases of osteomyelitis; however, may be challenging in delineating the gas within the bone. The other signs like bone marrow edema, cortical destruction, abnormal contrast enhancement and surrounding fluid collections can help accurately diagnose an infection and guide treatment.

A wide range of causative organisms have been isolated in pneumatic osteomyelitis including *Escherichia coli*, *Klebsiella pneumoniae*, *Enterobacter aerogenes*, *Clostridium *spp and anaerobes such as Anaerococcus, Peptostreptoccocus, Fusobacterium [9] etc. Both monomicrobial and polymicrobial infections have been reported [6].

The spread of infection is usually hematogenous, though contiguous spread from skin or soft tissue, or following orthopedic or intraabdominal surgery [10] have also been described in the literature.

EO is associated with significant morbidity and mortality, calculated at 34% in a review of available data from 41 cases [2]. The management usually rests on long course of antibiotic administration, including intravenous and oral, dictated by the culture sensitivity results. Combined medical and surgical treatment is often required. 

## Conclusions

EO is a rare infection that can be associated with a poor prognosis, especially in patients with underlying comorbidities. Outcomes in the past have often been fatal despite multiple serial surgical debridement procedures. Imaging plays a significant role in early diagnosis prompting physicians to initiate appropriate lifesaving management plan, including broad-spectrum antibiotics to cover anaerobes and surgical treatment where needed. 
